# Screen detection of ductal carcinoma in situ and subsequent incidence of invasive interval breast cancers: a retrospective population-based study

**DOI:** 10.1016/S1470-2045(15)00446-5

**Published:** 2016-01

**Authors:** Stephen W Duffy, Amanda Dibden, Dimitrios Michalopoulos, Judith Offman, Dharmishta Parmar, Jacquie Jenkins, Beverley Collins, Tony Robson, Suzanne Scorfield, Kathryn Green, Clare Hall, Xiao-Hui Liao, Michael Ryan, Fiona Johnson, Guy Stevens, Olive Kearins, Sarah Sellars, Julietta Patnick

**Affiliations:** a Policy Research Unit in Cancer Awareness, Screening and Early Diagnosis, Queen Mary University of London, Wolfson Institute of Preventive Medicine, London, UK; b Centre for Cancer Prevention, Queen Mary University of London, Wolfson Institute of Preventive Medicine, London, UK; c East Midlands QARC, Nottingham University Hospital City Campus, Nottingham, UK; d East of England QARC, Histon, Cambridge, UK; e London QARC, London, UK; f Yorkshire and Humber QARC, Newcastle upon Tyne, UK; g North West QARC, Albert St, Hollinwood, Oldham, UK; h Northern Ireland QARC, Belfast, UK; i South Central QARC, Oxford, UK; j South East Coast QARC, Battle, East Sussex, UK; k South West QARC, Clifton, Bristol, UK; l Public Health Wales, Cardiff, UK; m West Midlands QARC, Public Health Building, University of Birmingham, Birmingham, UK; n National Cancer Screening Programmes, Sheffield, UK

## Abstract

**Background:**

The value of screen detection and treatment of ductal carcinoma in situ (DCIS) is a matter of controversy. At present, the extent to which the diagnosis and treatment of DCIS could prevent the occurrence of invasive breast cancer in the future is not clear. We sought to estimate the association between detection of DCIS at screening and invasive interval cancers subsequent to the relevant screen.

**Methods:**

We obtained aggregate data for screen-detected cancers from 84 local screening units within 11 regional Quality Assurance Reference Centres in England, Wales, and Northern Ireland from the National Health Service Breast Screening Programme. Data for DCIS diagnoses were obtained for women aged 50–64 years who were invited to and attended mammographic breast screening from April 1, 2003, to March 31, 2007 (4 screening years). Patient-level data for interval cancer arising in the 36 months after each of these were analysed by Poisson regression with invasive interval cancer screen detection rate as the outcome variable; DCIS detection frequencies were fitted first as a continuous and then as a categorical variable. We repeated this analysis after adjustment with both small size and high-grade invasive screen-detected cancers.

**Findings:**

We analysed data for 5 243 658 women and on interval cancers occurring in the 36 months after the relevant screen. The average frequency of DCIS detected at screening was 1·60 per 1000 women screened (median 1·50 [unit range 1·54–3·56] per 1000 women). There was a significant negative association of screen-detected DCIS cases with the rate of invasive interval cancers (Poisson regression coefficient −0·084 [95% CI −0·13 to −0·03]; p=0·002). 90% of units had a DCIS detection frequency within the range of 1·00 to 2·22 per 1000 women; in these units, for every three screen-detected cases of DCIS, there was one fewer invasive interval cancer in the next 3 years. This association remained after adjustment for numbers of small screen-detected invasive cancers and for numbers of grade 3 invasive screen-detected cancers.

**Interpretation:**

The association between screen-detected DCIS and subsequent invasive interval cancers suggests that detection and treatment of DCIS is worthwhile in prevention of future invasive disease.

**Funding:**

UK Department of Health Policy Research Programme and NHS Cancer Screening Programmes.

## Introduction

The benefit-to-harm balance of screen detection and subsequent treatment of ductal carcinoma in situ of the breast (DCIS) has been a matter of debate.^1^, ^2^, ^3^ DCIS was rare in the era before screening, and a major question is the extent to which diagnosis and treatment of DCIS could prevent the occurrence of invasive breast cancer in the future.^4^ In two randomised trials of screening, an excess of DCIS in the study groups was almost entirely balanced by a corresponding deficit in invasive disease.^5^ Further, in a trial of treatment of DCIS, those cases who had only received wide local excision had a 10-year rate of subsequent breast cancer events of more than 30%, suggesting a serious potential of DCIS for progression.^6^ The fact remains, however, that for any individual DCIS case that is treated, it cannot be known for certain what would have happened if the treatment had not taken place.^6^ However, with sufficient aggregate data, it might be possible to assess the effect of screen detection of DCIS on the subsequent incidence of invasive cancer in a population subject to screening.

In this study, we sought to estimate the association between detection of DCIS at screening and invasive interval cancers subsequent to the relevant screen at unit level using data from the UK National Health Service Breast Screening Programme (NHSBSP).

## Methods

### Study population and design

The NHSBSP offers mammographic screening every 3 years to roughly 2 million women older than 50 years per year in the UK as a whole, and routinely invites women aged 50–70 years.^7^

**Research in context**

**Evidence before this study**

The value of screen detection and treatment of ductal carcinoma in situ (DCIS) is a matter of controversy. It is not clear the extent to which diagnosis and treatment of DCIS could prevent the occurrence of invasive breast cancer in the future.

We searched PubMed with the search terms “ductal carcinoma in situ” and “screening” and “breast” and “invasive” and “incidence” for publications reporting on studies of any design investigating the effect of screen detection of DCIS on incidence of subsequent invasive cancer, published in any language between Jan 1, 1990, and July 31, 2015. We identified a paper reporting on the effect of DCIS detection on invasive disease incidence in two randomised trials, but none from service screening programmes. The search was repeated with “ductal carcinoma in situ” replaced by “DCIS”, and no further relevant publications were identified.

**Added value of this study**

Using data from the UK NHS Breast Screening Programme, we estimated the effect of screen-detected DCIS on invasive interval cancers subsequent to the relevant screen at unit level. We found a negative association between detection of DCIS at screening and invasive interval cancers.

**Implications of all the available evidence**

This suggests that the policy of detection and treatment of DCIS in breast cancer screening can prevent subsequent invasive disease.

We obtained aggregate data for screen-detected cancers from 84 local screening units within 11 regional Quality Assurance Reference Centres in England, Wales, and Northern Ireland from the NHSBSP via the NHS Information Centre (now the Health and Social Care Information Centre [HSCIC]) and its counterparts in Northern Ireland and Wales. Data for breast cancer diagnoses, including DCIS, were obtained for women aged 50–64 years (age range of the programme at its inception) who were invited to and attended mammographic breast screening from April 1, 2003, to March 31, 2007 (4 screening years). Patient-level data for interval cancer were obtained from the regional screening Quality Assurance Reference Centres, who in turn received notifications of subsequent interval cancers arising in the 36 months following screening from their regional cancer registries, for the number of subsequent interval cancers arising in the 36 months after each of these screening years. Interval cancer data for Scotland was unavailable for all but one of the screening years so this region was excluded from the primary analysis. Where regional boundaries changed during the period under scrutiny, configurations of regions and responsibilities for units were taken to be those that existed in screening year 2006–07.

### Procedures

From the aggregate data, we were able to ascertain the proportion of women diagnosed with DCIS and invasive tumours. When available we obtained DCIS grade and invasive tumour size and grade**;** DCIS tumours are graded as low, intermediate, and high, with low and intermediate grades grouped together. Usually, DCIS is treated with complete local excision followed by radiotherapy; however, the NHSBSP data are recorded mainly to monitor performance and thus do not contain information regarding subsequent treatment of patients with screen-detected cancer. As in a previous report,^8^ interval cancers were defined as cancers diagnosed symptomatically in women within 36 months of their last screen (the maximum specified interval in the NHSBSP). For this analysis, we included first and second primary, bilateral, and recurrent invasive interval cancers, but excluded contralateral interval cancers to ensure the outcome variable consisted of new primary breast cancers and because it is unlikely that the removal of a DCIS in one breast could prevent an invasive tumour in the contralateral breast.

### Statistical analysis

For our primary analysis, we estimated the association between the frequency of DCIS cases diagnosed at screening and subsequent invasive interval cancer incidence using Poisson regression,^9^ with the following regression equation:

$$\log\left(\frac{\text{i}}{\text{s}}\right)=\text{a}+\text{b}\times \log\left(\frac{\text{D}}{\text{s}}\right)$$

where i represents invasive interval cancers in the individual screening unit following screening in an individual year, s is the total number screened in that unit and year, D is the number of screen-detected DCIS cases in that unit and year, and a and b are the coefficients to be estimated. In addition, we tabulated the incidence of invasive interval cancers by DCIS detection frequency in four categories (<1 per 1000; 1–<1·5 per 1000; 1·5–<2 per 1000; and ≥2 per 1000), and fitted the corresponding Poisson regression model. This allowed us to estimate the absolute deficit or increase in terms of the number of invasive interval cancers pertaining to different frequencies of DCIS, without assuming a parametric form for the relationship. In addition, we fitted the Poisson regression models adjusted for the number of screen-detected grade 3 and small (<15 mm in diameter) invasive cancers, because there is evidence that high numbers of small invasive tumours detected at screening are associated with lower interval cancer incidence.^10^ Significance was assessed with Wald tests, with p values of less than 0·05 being regarded as significant. Because the average interval between screening rounds varies from unit to unit (although most units achieve an average interval of less than 36 months), we also reanalysed the data with only the interval cancers diagnosed within the first 24 months following a negative screen, since no unit operated an interval shorter than this, and because 24 months is the standard screening interval in many other countries.^2^, ^10^ We also repeated the analysis to include the data available from Scotland for screening year 2003–04. Data were analysed with Stata (version 12).

### Role of the funding source

The funders had no role in the study design; collection, analysis, or interpretation of the data; or the writing of the report. SWD, AD, DM, JO, and DP had full access to all of the raw data. The corresponding author had the final responsibility for the decision to submit for publication.

## Results

We obtained aggregated screening data for 5 243 658 women screened between April 1, 2003, and March 31, 2007. The number of women screened by each screening unit each year is shown in the appendix. One unit in the South West region only began screening in 2004–05 and thus we have data covering three screening years for this unit. Another unit in the East of England suspended screening between November, 2004, and January, 2006. Subsequently, only 550 women were screened during screening year 2005–06. All analyses were done both including and excluding these women; the results were identical so we present the results excluding data of these 550 women from the analysis.

The average number of women screened annually per unit was 15 700 (unit range 3835–40 146). Table 1 shows the incidence of screen-detected DCIS and screen-detected invasive cancers for each screening year, and the incidence of subsequent invasive interval cancers. The average frequency of DCIS detected at screening was 1·60 per 1000 women screened (median 1·50 [unit range 0·54–3·56] per 1000). Among DCIS cases that were graded, there was a higher proportion of low-grade DCIS in units with a detection frequency higher than 1·5 per 1000 than in those units with a detection frequency of 1·5 per 1000 or less (1866 [40%] of 4663 *vs* 967 [38%] of 2569; p=0·008). The average frequency of screen-detected invasive cancers was 5·53 per 1000 women screened (unit range 0–7·99). The subsequent average detection frequency of invasive interval cancers was 2·90 per 1000 screened (unit range 0·74–5·60 per 1000).

The primary analysis to estimate the association between screen-detected DCIS incidence and subsequent invasive interval cancer incidence resulted in a Poisson regression coefficient of −0·084 (95% CI −0·13 to −0·03), a significant negative association (p=0·002). The Poisson regression coefficient is equivalent to the logarithm of the relative risk (RR), thus a coefficient of −0·084 indicates a reduction in risk of an invasive interval cancer per unit increase in the logarithm of the detection frequency of DCIS (RR 0·92 [95% CI 0·87–0·98]). The fitted incidence of invasive interval cancers per 1000 from the Poisson regression plotted against the frequency of screen-detected DCIS per 1000 is shown in the figure. For up to around 1·5 per 1000 women screened, there are estimated two fewer invasive interval cancer cases for every three DCIS cases; from 1·5 to 2·5 per 1000, around one invasive interval cancer case is estimated to be avoided per five DCIS cases. When the analyses were adjusted for grade 3 invasive cancers or small invasive cancers (<15 mm) the results remained unchanged from the primary analysis (table 2). We repeated this analysis after restricting interval cancers to those diagnosed within 24 months after a negative screen and after including the 1 year of data available from Scotland. Results of the Poisson regressions in relation to the magnitude and significance of the Poisson regression coefficient were similar to that of the primary analysis (table 2).

To estimate the absolute change in invasive interval cancers corresponding to different detected frequencies of DCIS without assuming a specific mathematical form of the association, we fitted the model with DCIS detection frequencies as a categorical variable (table 3). These data suggest that at a DCIS detection frequency of 1–1·5 per 1000, for two DCIS cases detected at screening, one invasive cancer in the interval immediately following the screen was avoided (a difference of 3·15–2·94=0·21 per 1000 invasive interval cancers compared with 1·27–0·85=0·42 per 1000 screen-detected DCIS). For units with DCIS detection rates above 1·5 per 1000, there was a reduction of one invasive interval cancer per six DCIS cases detected. 90% of units had a DCIS frequ within the range of 1·00 to 2·22 per 1000; within this range, roughly one fewer invasive interval cancer was noted per three screen-detected DCIS cases (figure).

## Discussion

Using data from the UK NHSBSP we identified a significant negative association between the number of DCIS cases detected at screening and the number of invasive cancers occurring in the subsequent 3-year interval. To our knowledge, this is the first study to explicitly investigate the association between screen detection of DCIS and subsequent incidence of invasive breast cancer. Previous research has suggested that the frequency of detection of small invasive cancers might be negatively correlated with subsequent interval cancer incidence^10^ and increased detection of DCIS has been associated with similarly increased detection of grade 3 invasive cancers.^11^ Our results were unchanged when we adjusted for the detection of small invasive cancers and when we adjusted for the detection of grade 3 invasive cancers.

Our results suggest that at low levels of screen-detected DCIS, up to 1·5 per 1000, a reduction of one invasive interval cancer is observed per 1·5–3 DCIS cases detected, and that at higher levels of detection, one less invasive interval cancer is observed per five or six screen-detected DCIS cases. One possible interpretation is that there might be diminishing returns with more aggressive diagnostic approaches to microcalcifications, resulting in high detection frequencies of DCIS. On the other hand, it could be that detection of DCIS above two per 1000 prevents invasive cancers more than 3 years in the future, and that our study of the single interval after screen detection is too short to observe this. We noted that the units with increased detection of DCIS overall had increased proportions of low-grade DCIS, which might be expected to progress to invasive disease over a time period longer than 3 years. For units with DCIS screen-detection frequencies that fall within the 90% range for all units, on average one invasive interval cancer would be avoided per three additional DCIS cases detected.

Our results are ecological, based on screening unit level data. This means, for example, that we cannot link DCIS cases detected at screening with invasive interval cancers at the individual level. We have also made no adjustment for age, although we would not anticipate major variation among units within the relatively narrow window of 50–64 years. Both DCIS screen-detection frequency and invasive interval cancer frequency would be expected to increase slightly with age.^7^, ^8^ Our findings cannot give definitive proof of the progressive potential or otherwise of individual DCIS cases. Only the measure of leaving DCIS untreated could give such evidence. Our results do, however, add to the evidence that detection and treatment of DCIS is worthwhile in the prevention of future invasive disease.^5^, ^6^, ^12^, ^13^, ^14^ It is also worth noting that our results are consistent with a review of interval cancer incidence from eight screening rounds within four screening programmes, which tabulated invasive and in-situ screen-detection frequency with the interval cancer incidence.^15^ If one calculates the crude correlation coefficient of the in-situ screen-detection frequency with the interval cancer incidence reported in table 4 of that paper, a negative correlation of −0·29 is obtained.^15^

The median frequency of DCIS detected at screening was 1·5 per 1000 women screened, ranging from 0·5 to 3·6 per 1000. Several factors affect the frequency of screen-detected DCIS and invasive cancer incidence, including age distribution among screening units, varying underlying cancer incidence, experience of the relevant professionals, and equipment differences. Cancers identified in the screening programme reports as non-invasive were classed as DCIS, thus these will contain a small proportion of microinvasive cancers.

The NHSBSP screens women in the age range of 50–70 years.^7^ When the programme began in 1988, the target age range was 50–64 years, but was extended to include woman up to the age of 70 years during 2003–05 in England and Wales and in 2010 in Northern Ireland. In England, the age range is being expanded to 47–73 years, mostly as part of a randomised trial (ISRCTN33292440). However, there is ongoing and highly publicised debate about the harm caused by mammography screening in regard to overdiagnosis.^16^, ^17^, ^18^ Although it cannot be known for certain what would happen for any individual DCIS that is treated, our findings suggest that the treatment of DCIS is worthwhile in the prevention of subsequent invasive disease.

We intend to do similar analyses to relate invasive and DCIS detection incidence from the screening data to those from the next scheduled screen of the same cohorts 3 years later. An analysis of invasive cancers in the breast screening programme in Scotland noted that a high frequency of small invasive tumours at the prevalence screen was predictive of a low frequency of large invasive tumours at the incidence screen 3 years later, although this is confounded by number of mammographic views.^19^

In conclusion, we have found a negative association between screen detection of DCIS and subsequent invasive interval cancer incidence. Up to the median DCIS detection rate of 1·5 per 1000, we observed roughly one fewer invasive interval cancer per 1·5–3 DCIS cases.

## Figures and Tables

**Figure:**
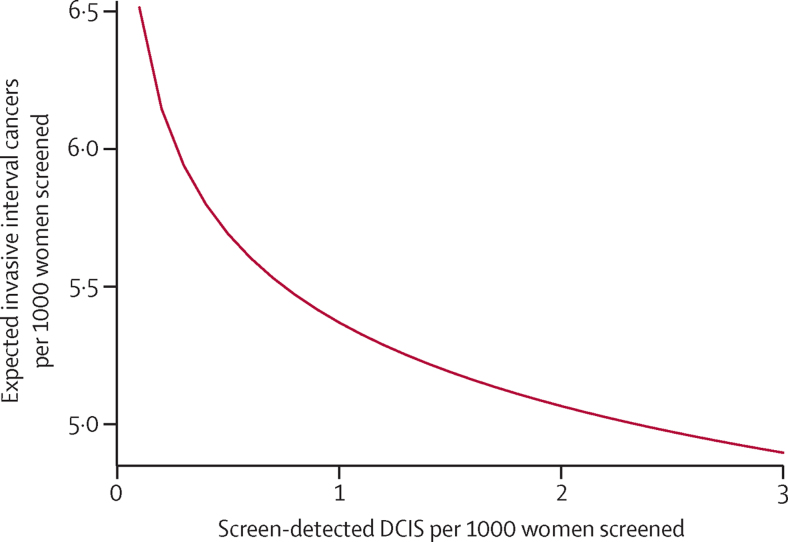
Fitted invasive interval cancer incidence from Poisson regression as a function of DCIS detection frequency at the previous screen DCIS=ductal carcinoma in situ.

**Table 1:** DCIS and invasive cancer detection frequency, and subsequent invasive interval cancer frequency by year of screening

	**Total number screened**	**DCIS cases detected at screening**	**Screen-detected DCIS cases per 1000 women screened (unit range)**	**Screen-detected DCIS cases with grading information (%)**	**High grade screen-detected DCIS**	**Invasive cases detected at screening**	**Invasive screen-detected cases per 1000 women screened (unit range)**	**Invasive interval cancers**	**Invasive interval cancers per 1000 women screened (unit range)**
2003–04	1 279 007	2003	1·57 (0·73–2·85)	1671 (83%)	997	6952	5·44 (0–7·61)	3789	2·96 (1·00–4·96)
2004–05	1 286 244	2033	1·58 (0·67–3·56)	1708 (84%)	1036	7194	5·59 (3·74–7·99)	3688	2·87 (0·74–4·05)
2005–06	1 340 046	2144	1·60 (0·54–2·85)	1842 (86%)	1119	7504	5·60 (3·85–7·64)	4000	2·98 (1·27–5·60)
2006–07	1 338 361	2205	1·65 (0·59–3·22)	1956 (89%)	1213	7364	5·50 (3·77–7·77)	3737	2·79 (1·25–4·61)

DCIS=ductal carcinoma in situ.

**Table 2:** Results of secondary analyses of association of DCIS detection frequencies with subsequent invasive interval cancer incidence

	**Poisson regression coefficient (95% CI)**	**p value**
**Adjustment for small (<15 mm) invasive tumours**		
Frequency of screen-detected DCIS	−0·080 (−0·13 to −0·03)	0·003
Small screen-detected tumours	−0·0003 (−0·001 to 0·0004)	0·38
**Adjustment for screen-detected grade 3 invasive tumours**		
Frequency of screen-detected DCIS	−0·089 (−0·14 to −0·03)	0·001
Grade 3 screen-detected tumours	0·0003 (−0·0003 to 0·001)	0·35
**Restriction to invasive interval cancers diagnosed within 24 months**		
Frequency of screen-detected DCIS	−0·089 (−0·16 to −0·016)	0·016
**Repeat of primary analysis to include the 2003–04 data from Scotland**		
Frequency of screen-detected DCIS	−0·086 (−0·14 to −0·033)	0·001

DCIS=ductal carcinoma in situ.

**Table 3:** Association of categorised DCIS detection frequencies at screening with subsequent invasive interval cancer incidence

	**Average DCIS detection per 1000**	**Invasive interval cancers/total screened**	**Invasive interval cancers per 1000**	**RR (95% CI)**
<1 per 1000	0·85	1236/392 982	3·15	1·0
1–<1·5 per 1000	1·27	6069/2 063 030	2·94	0·94 (0·88–0·99)
1·5–<2·0 per 1000	1·72	4844/1 686 588	2·87	0·91 (0·86–0·97)
≥2 per 1000	2·25	3065/1 101 058	2·78	0·89 (0·83–0·95)

DCIS=ductal carcinoma in situ. RR=relative risk.
