# Correction to *Lancet Oncol* 2016; 17: 109

**DOI:** 10.1016/S1470-2045(16)00021-8

**Published:** 2016-02

*Duffy SW, Dibden A, Michalopoulos D, et al. Screen detection of ductal carcinoma in situ and subsequent incidence of invasive interval breast cancers: a retrospective population-based study.* Lancet Oncol *2016; **17:** 109–14*—In the Findings section of the Summary of this Article, the second sentence should read “The average frequency of DCIS detected at screening was 1·60 per 1000 women screened (median 1·50 [unit range 0·54–3·56] per 1000 women).” This correction has been made to the online version as of Feb 2, 2016.

