# Supplementary material for: Screen detection of ductal carcinoma in situ and subsequent incidence of invasive interval breast cancers: a retrospective population-based study
Source: Lancet Oncol. 2016 Jan;17(1):109–14. doi: 10.1016/S1470-2045(15)00446-5 (PMC4691349; doi:10.1016/S1470-2045(15)00446-5)
Supplement: Supplementary appendix [file mmc1.pdf]

THE LANCET Oncology

Supplementary appendix

This appendix formed part of the original submission and has been peer reviewed. We post it as supplied by the authors.

Supplement to: Duffy SW, Dibden A, Michalopoulos D, et al. Screen detection of ductal carcinoma in situ and subsequent incidence of invasive interval breast cancers: a retrospective population-based study. *Lancet Oncol* 2015; published online Dec 4. http://dx.doi.org/10.1016/S1470-2045(15)00446-5.

Supplementary Material

Table 1: Number of women screened by screening unit and year

Screening Unit	Total number of women screened by screening year			
	2003/04	2004/05	2005/06	2006/07
AGA	19263	22808	18953	21674
ANE	19147	21282	22810	22452
ANT	18025	24449	25088	22373
AWC	8385	9879	9292	9029
BHL	23271	20909	19515	16479
BHU	22582	26199	27036	24665
BLE	22634	19627	23598	19741
BYO	18621	20035	21575	21870
CBA	6331	5331	6956	6115
CDN	9894	9136	10477	10232
CDO	10154	14987	8270	10388
CDS	15955	14486	16394	15620
CLE	22908	23666	25345	25615
CLI	16760	17342	15624	15030
CNN	5670	6615	6105	6285
CNO	16366	15519	20434	18747
CRO	6505	7008	6657	7161
CSH	12493	10459	11415	12365
DCB	13521	10402	9173	14899
DGY	6878	4815	7999	7228
DKL	6650	4609	6543	6371
DNF	13271	14223	18303	14248
DPT	8405	6959	9862	8080
DSU	9991	11193	8577	9681
DSW	6918	7431	9498	5225
EBA	29135	28851	30250	23342
ECX	21161	21997	25979	26300
ELD	30351	28916	30714	33249
FBH	11391	9745	13352	17903
FCO	16525	17571	20202	18255
FEP	11529	6775	Excluded[b]	6931
FLO	16331	15051	16758	15868
FSO	15590	15317	15760	15212
GBR	20064	19807	19252	17620
GCA	27742	26743	28215	29755
GCT1	15181	16545	10174	17069
GCT2	10317	10991	17374	11979
GCT3	13411	13027	13650	16357
HGU	29029	29953	31478	31424
HWA	23520	25468	23822	28464
HWO	16496	19431	21952	19508

JBA	10762	12758	13403	11947
JDO	20292	18383	19305	20746
JIW	3835	3979	5374	4149
JPO	14665	10116	12522	16376
JSO	14333	15726	14497	16109
JSW	13031	13385	13003	14022
KHW	15709	15265	13140	13523
KKE	7931	8866	9591	8038
KMK	5448	6254	6146	6822
KNN	10588	9390	9133	9829
KOX	16205	12153	14586	14123
KRG	10801	12698	11479	11670
KWI	10753	10007	11144	11045
LAV	21018	19706	24181	30978
LCO	13292	13156	12547	10676
LED	11925	10961	15975	15763
LGL	16738	14500	14909	17342
LPL	15480	14029	15064	14446
LSO	12443	14867	13282	13237
LTB	N/A[a]	7255	9751	6789
MAS	12479	16766	19337	18577
MBS	7298	8121	9416	7596
MBW	21955	22397	23411	22402
MCO	25584	25687	27415	25458
MDU	12296	13904	14389	11972
MHW	24693	22500	18776	22926
MSH	10484	13701	10375	14160
MST	10987	11139	13064	11786
NCH	4238	6166	4438	4671
NCR	8731	7022	8065	8887
NI	38926	30146	32061	31584
NLI	16428	19398	19408	17057
NMA	11712	12547	11927	13152
NWA	15120	13466	12697	14402
NWI	10984	8589	8348	10411
PBO	15528	15355	16023	16950
PLE	11420	12641	12259	10599
PLN	18251	17475	17482	16127
PMA	25340	22283	20610	27180
PWI	15469	16998	15243	15341
WNM	16130	15852	20820	15686
WSE	35509	33521	40146	35912
WSW	15825	21559	20873	17086

[a.] LTB began screening in 2004/05

[b.] Screening suspended between November 2004 and January 2005 so data for this unit for screening year 2004/05 was excluded from the analysis
